# Predictors of outcome in childhood *Plasmodium falciparum* malaria

**DOI:** 10.1080/21505594.2020.1726570

**Published:** 2020-02-17

**Authors:** Harsita Patel, Claire Dunican, Aubrey J. Cunnington

**Affiliations:** Section of Paediatric Infectious Disease, Department of Infectious Disease, Imperial College London, London, UK

**Keywords:** Severe malaria, cerebral malaria, severe malarial anemia, respiratory distress, prognosis, outcome prediction, molecular biomarkers, metabolic acidosis, childhood malaria, *Plasmodium falciparum*

## Abstract

*Plasmodium falciparum* malaria is classified as either uncomplicated or severe, determining clinical management and providing a framework for understanding pathogenesis. Severe malaria in children is defined by the presence of one or more features associated with adverse outcome, but there is wide variation in the predictive value of these features. Here we review the evidence for the usefulness of these features, alone and in combination, to predict death and other adverse outcomes, and we consider the role that molecular biomarkers may play in augmenting this prediction. We also examine whether a more personalized approach to predicting outcome for specific presenting syndromes of severe malaria, particularly cerebral malaria, has the potential to be more accurate. We note a general need for better external validation in studies of outcome predictors and for the demonstration that predictors can be used to guide clinical management in a way that improves survival and long-term health.

## Introduction

Malaria is a mosquito-transmitted infectious disease caused by protozoan parasites of the genus *Plasmodium*. Over 150 *Plasmodium* species have been identified [1] and found to infect a diverse range of hosts from birds and reptiles to deer and monkeys. Infections in humans primarily involve five *Plasmodium* species: *Plasmodium falciparum, P. vivax, P. ovale, P. malariae*, and *P. knowlesi*, although several other species have been observed to cause zoonotic infections.

According to the World Health Organization (WHO), there were 219 million reported cases of malaria across 87 countries in 2017. Countries within the WHO Africa region had the highest incidence and accounted for approximately 92% of malaria cases and 93% of malaria deaths. This corresponds to an estimated 435,000 deaths directly caused by malaria in the same year, the majority (61%) being in children under five [2]. This review will specifically focus on predictors of death and other adverse outcomes in children with malaria.

Many factors can influence the clinical outcomes of malaria, including parasite species, host, and parasite genetics, innate and acquired immunity, access to appropriate treatment, comorbidities, and antimalarial resistance. *P. falciparum* accounts for the vast majority of severe malaria (SM) and malaria deaths globally, and is the focus of this review, but *P. vivax* and *P. knowlesi* can also cause severe and fatal infections, whilst *P. malariae* and *P. ovale* almost always cause uncomplicated malaria (UM) [3,4]. In the case of *P. falciparum*, naturally acquired immunity develops with repeated infections in high transmission settings resulting in the sequential acquisition of protection from severe disease and then from symptomatic infection [5]. In these regions, young children who have not yet acquired immunity are at greatest risk of life-threatening malaria, whereas all age groups typically remain susceptible in the lowest transmission settings where exposure is insufficient to induce immunity [6].

The life cycle of *P. falciparum* involves two hosts, the female *Anopheles* mosquito and the human. Unlike the human host, the mosquito suffers no deleterious effects of the infection. In humans, the infection starts when the sporozoite form of the parasite enters the skin during mosquito feeding and then rapidly transits through the bloodstream to the liver where there is a pre-symptomatic incubation period of replication within hepatocytes [7]. After about 1 week, the parasites leave the liver and enter the bloodstream where they invade red blood cells and undergo repeated 48-h cycles of asexual replication [5]. These asexual blood-stage parasites are the cause of symptoms that define the onset of malaria [8]. Some parasites will also differentiate into sexual stage gametocytes, ready for uptake by another mosquito bite and continuation of the transmission cycle, but gametocytes themselves do not cause any clinical symptoms [7].

Like most other infectious diseases, the consequences of infection with *P. falciparum* can vary widely between individuals, and the observed severity is also influenced by how soon after infection an individual is assessed and treated. Outcomes of infection can range from asymptomatic carriage of parasites (seen in individuals with substantial naturally acquired immunity) to unpleasant influenza-like symptoms, to significant organ dysfunction and death [9]. In this review, we focus only on symptomatic infections. UM is defined by the presence of asexual blood-stage parasites and typical clinical symptoms of malaria such as fever, headache, nausea, and vomiting in the absence of another cause and without the features which define SM [9]. SM is defined by similar criteria except that one or more clinical or laboratory features associated with increased risk of death is also present (Table 1).

**Table 1.** Clinical and laboratory features of severe malaria and their prognostic value.

Clinical feature	Definition	Frequency (± to +++)	Prognostic Value (± to +++)	Range of effect size [95% CI])
Impaired Consciousness	Glasgow Coma Score < 11 or Blantyre score < 3 in children who are too young to speak	+++	+++	Marsh et al., [10] RR 2.00 [0.90–4.10] Leopold el al., [73] RR 4.38 [3.75–5.12] (“coma”) Jallow et al., [29] RR 4.50 [3.60–5.60] Marsh et al., [10] RR 12.6 [7.20–22.0] (“coma”, defined as when patients are unable to locate painful stimuli) Evans et al., [71] RR 13.5 [CI not stated]
Respiratory Distress	Rapid, deep and labored breathing or oxygen saturations < 92%	+++	+++	Waller et al., [69] RR 2.70 [1.20–5.80] (abnormal chest signs) Jallow et al., [29] RR 3.40 [2.80–4.20] Evans et al., [71] RR 3.91 [2.83–5.41] Maitland et al., [68] OR 5.10 [2.90–9.00] Gérardin et al., [234] OR 7.44 [2.82–21.7] Marsh et al., [10] RR 9.40 [5.50–16.2] Njuguna et al., [23] OR 9.41 [7.69–11.5] Waller et al., [69] RR 11.4 [4.00–33.0] (pulmonary complications)
Multiple Convulsions	2 or more seizures within 24 hours	+++	+	Jallow et al., [29] RR 2.0 [1.50–2.50] Njuguna et al., [23] OR 2.21 [1.67 to 2.92] Marsh et al., [10] RR 2.90 [1.70–4.90] Gérardin et al., [234] OR 3.01 [1.14–7.58]
Prostration	Inability to sit unassisted or feed (in babies) due to extreme weakness	+++	+	Njuguna et al., [23] OR 1.05 (0.71 to 1.56) Marsh et al., [10] RR 1.80 [0.90–3.60] Waller et al., [69] RR 2.40 [1.10–5.00] Evans et al., [71] RR 3.15 [1.67–5.95] Jallow et al., [29] RR 3.70 [1.50–9.00]
Shock	Compensated: capillary refill time > 3s or temperature gradient from mid to proximal limb Decompensated: systolic blood pressure < 70mmHg	+	+++	Evans et al., [71] RR 2.26 [1.63–3.13] Jallow et al., [29] RR 2.80 [1.00–8.30] Maitland et al., [68] OR 2.80 [1.50–5.20] Njuguna et al., [23] OR 4.76 [3.40 to 6.66]
Pulmonary Edema	Radiologically confirmed or oxygen saturation <92% in air with respiratory rate >30/min, may be associated with clinical wheeze/crepitations	±	+++	Gérardin et al., [234] OR 0 [0.00–54.43] Leopold el al., [73] 4.54 (95% CI 3.07–6.71)
Significant Bleeding	Including recurrent or prolonged bleeding from nose, gums or venepuncture sites; blood in vomit or stool	±	+++	NA
Jaundice	Plasma or serum bilirubin >50 µM with a parasite count >10,000/µl	+	++	Jallow et al., [29] RR 1.60 [1.20–2.10] Njuguna et al., [23] OR 2.43 [1.49 to 3.98] Gérardin et al., [234] OR 2.69 [0.99–6.88] Marsh et al., [10] RR 4.60 [2.20–9.40]
Severe Malaria Anemia	Hemoglobin < 5g/dL or hematocrit < 15%	+++	+	Evans et al., [71] RR 0.37 [0.28–0.50] Jallow et al., [29] RR 0.50 [0.40–0.70] Marsh et al., [10] RR 1.40 [0.80–2.50] Gérardin et al., [234] OR 1.45 [0.40–4.27] Maitland et al., [68] OR 1.70 [1.00–3.10] Obonyo et al., [235] RR 2.04 [1.32–3.16] Njuguna et al., [23] OR 2.24 [1.94–2.60] Waller et al., [69] RR 3.40 [1.60–7.10]
Hypoglycemia	Blood or plasma glucose < 2.2mM	+++	+++	Jallow et al., [29] RR 1.80 [1.40–2.30] Gérardin et al., [234] OR 2.97 [0.89–8.64] Maitland et al., [68] OR 3.40 [1.70–6.70] Evans et al., [71] RR 4.25 [3.09–5.83] Marsh et al., [10] RR 5.40 [2.90–10.2] Waller et al., [69] RR 7.10 [3.60–13.8] Njuguna et al., [23] OR 8.81 [6.98 to 11.1]
Metabolic Acidosis	Base deficit of >8 mEq/L or plasma bicarbonate of <15 mM.	+++	+++	Marsh et al., [10] RR 2.10 [0.60–7.00] Maitland et al., [68] OR 2.30 [1.20–4.40] Jallow et al., [29] RR 5.60 [2.40–12.9] Gérardin et al., [234] OR 5.94 [2.28–15.1] Njuguna et al., [23] OR 7.07 [5.77–8.67]
Hyperlactataemia	Plasma or whole blood lactate > 5mM	+++	+++	Jallow et al., [29] RR 1.30 [0.80–2.20] Evans et al., [71] RR 1.37 [0.74–2.54] (lactate 5.0–6.9 mmol/L) Evans et al., [71] RR 2.60 [1.40–4.81] (lactate 7.0–8.9 mmol/L) Evans et al., [71] RR 6.28 [3.74–10.6] (lactate ≥ 9 mmol/L)
Renal Impairment	Plasma or serum creatinine >265µM (3 mg/dl) or blood urea >20 mM	+	++	Maitland et al., [68] OR 3.00 [1.70–5.40] (creatinine >80 μmol/L) Jallow et al., [29] RR 5.10 [3.20–8.00] Njuguna et al., [23] OR 6.09 [4.43–8.37] (kidney injury) Gérardin et al., [234] OR 11.3 [1.40–87.4]
Hyperparasitaemia	*P. falciparum* parasitemia >10% in peripheral blood	++	±	Gérardin et al., [234] OR 0.79 [0.14–2.83] (parasitemia≥4%) Njuguna et al., [23] OR 1.19 [1.02–1.38] Evans et al., [71] RR 1.37 [0.90–2.08] Marsh et al., [10] RR 1.40 [0.60–3.00] Jallow et al., [29] RR 1.60 [1.10–2.30] Waller et al., [69] children with hyperparasitaemia (parasite count ≥ 500, 000/µL) were 2.20 [1.10–4.40] times more likely to die.
Thrombocytopenia	Platelet levels < 20,000/µL	NA	NA	Gérardin et al., [234] OR 6.13 [1.70–32.8] (platelet count < 100,000/mm^3^) Lampah et al., [236] OR 11.3 [9.49–13.4] (platelet count < 50,000 g/dL)

**Legend**

The definition, frequency, and prognostic value of clinical and laboratory features of SM in children under 12 y of age are indicated semi-quantitatively from equivocal (±) to high (+++), based on “Severe Malaria” Review published by the World Health Organization in Tropical Medicine and International Health 2014 SM [17]. Effect sizes and confidence intervals are shown for studies that analyzed the association of any severity feature with death without adjustment for the presence of other severity features and quantified the effect size. Studies where this was unclear have been excluded from the table. Abbreviations: Confidence Interval (CI); Odds Ratio (OR); Relative Risk (RR)

For many laboratory and clinical parameters, the distinction between SM and UM is determined by a threshold value and it is important to note that these thresholds are somewhat artificial for the convenience of classification. For example, hemoglobin (Hb), hematocrit, blood glucose, and lactate levels are all continuous variables that are likely to have a continuous relationship with the risk of death rather than a step-effect at a particular threshold value. It is also important to recognize that SM is a multisystem, multi-organ disease, although SM in children is often categorized into three easily recognized clinical syndromes that can occur separately or in combination: cerebral malaria (CM), severe malarial anemia (SMA), and respiratory distress [10,11].

A substantial amount of work has been conducted to understand what distinguishes the pathogenesis of different SM phenotypes from UM and from each other with the hope of identifying targets for adjunctive therapy. Unfortunately, to date, no adjunctive treatments have proven effective in clinical trials [12]. Similar comparisons have also indicated molecular biomarkers that distinguish between SM and UM. Whilst this in itself is not necessarily useful when SM is already classified based on clinical features and routine laboratory tests, it is reasonable to expect that some biomarkers associated with the occurrence of SM might also predict the risk of death or long-term morbidity.

Prognostic markers for mortality and long-term morbidity could have several applications. Foremost, they could help to determine the most appropriate course of action for each individual patient by informing the choice of medical treatment and whether transfer to a medical facility with higher-level care facilities is required. Secondarily they may be used as inclusion criteria, or for stratification, in clinical trials of adjunctive treatments or interventions to improve outcomes in SM, potentially increasing power to detect effects on the outcome. Third, identification of children with greatest risk of long-term sequelae could potentially be used to target interventions to reduce the impact of these sequelae on their life, even if the sequelae cannot be prevented.

In this literature review, we critically evaluate the usefulness of clinical and laboratory features of SM as predictors of mortality and long-term complications in childhood *P. falciparum* malaria and assess the potential of molecular biomarkers and other approaches to improve the accuracy of prediction. We consider these questions for SM in general and for the major syndromes of CM, SMA, and respiratory distress individually. We briefly consider the important role of coinfections (human immunodeficiency virus (HIV) and bacteremia) as well as malaria in returning travelers. Finally, we synthesize the existing evidence and propose strategies to improve outcome prediction in pediatric *P. falciparum* malaria.

Publications considered in this review were identified through keyword searches of the PubMed database using terms specific to each sub-section of the review. Additional publications were identified from our own bibliographies and from references in articles identified through Pubmed. We did not exclude any studies based on study size, and thus studies ranged in size from tens to thousands of patients. The largest study, from the Severe Malaria in African Children (SMAC) network included data from 20,333 patients collected between 2000 and 2003 from 5 sites (Banjul, Blantyre, Kilifi, Kumasi, and Lambarene) [13]. Wherever possible we report confidence intervals for estimates of risk or association, which indicate the precision of these estimates, in part determined by the study size.

## Prognostic value of criteria defining severe malaria

The criteria commonly used to define SM were selected because they are associated with adverse outcome, however, they are not all equally good predictors (Table 1) and the evidence for each will be discussed in more detail below.

One important general caveat is that interpretation of the relationship of prognostic indicators with outcomes is often complicated by the difficulty of knowing whether malaria is the primary or only cause of these features. In some high transmission settings, it is very common for parasitemia to be an incidental finding in children with other febrile illnesses with similar presentations. For example, a child with decreased conscious level due to bacterial meningitis who has incidental parasitemia might be classified as having CM unless suitable diagnostic facilities are available to identify the meningitis. In this case, the prognosis would be more dependent on appropriate treatment of the meningitis than of the malaria parasites.

Another caveat is that direct comparison of the Relative Risk (RR), Hazard Ratio (HR), Odds Ratio (OR), and Adjusted Odds Ratio (AOR) for death between studies requires some caution. Variation in study size and design, different study settings, different populations, and variation in the antimalarial and supportive care treatments available, likely contribute to the heterogeneity of the risk estimates. For example, the increasing use of intravenous artesunate over the last decade has reduced the likelihood of death in SM compared with studies conducted in the pre-artesunate era. Data originating from clinical trials may show lower overall and relative risks of mortality than in observational studies, an effect often attributed to improved clinical care provided during clinical trials. This is particularly evident in the “Artesunate versus quinine in the treatment of severe *falciparum* malaria in African children trial (AQUAMAT)” by von Seidlein et al. [14], which showed consistently lower risks of death associated with severity features than other studies examining the same clinical features.

### Hyperparasitaemia

In many infectious diseases, pathogen load is thought to be linked to outcome [15]. In malaria, high parasitemia can indicate a high parasite replication rate, insufficient clearance of parasites by the host response, and or a longer duration of infection [16]. Peripheral hyperparasitaemia (Table 1) is considered an important indicator of SM [17,237]. The definition of hyperparasitaemia is dependent on the setting: in low prevalence settings and returning travelers the threshold defining SM is often set at 4–5% of red blood cells [18,19], whereas in areas of stable endemicity the WHO recommends >10% [17,20].

Hyperparasitaemia has been associated with an increased likelihood of symptomatic malaria infections [21] as well as malaria-associated mortality [22]. In multivariable analyses of the SMAC cohort, hyperparasitaemia was associated with mortality (OR of 3.6 [95% CI 1.8–7.5]) in Banjul (The Gambia), but not at the other four study sites [22], and other studies have also found that its prognostic significance diminished when it was combined with other prognostic markers [14,23]. Interestingly, in a study of Kenyan children admitted to hospital over a 27-y period during which the incidence of SM declined dramatically, hyperparasitaemia (defined as parasite density >250,000 parasites per μl) only emerged as a predictor of death during the lowest incidence period [23]. This suggests that the effect of hyperparasitaemia on outcome may only be apparent at lower transmission intensities. It is also important to note that circulating parasitemia is a poor indicator of total body parasite load in *P. falciparum* malaria because the late asexual blood stage parasites sequester in the microcirculation and are not usually sampled in the blood used to estimate parasitemia [24]. For this reason, it is not surprising that hyperparasitaemia is a relatively poor predictor of outcome, as illustrated in Table 1 by the low RR values and low prognostic value given by the WHO. Indeed, Silamut & White showed that when these late blood-stage parasites are visible in the circulation it is a grave prognostic marker [25].

### Hypoglycemia

Hypoglycemia (Table 1) has been reported in 1–32% of children presenting to hospital with malaria [26–30], with a greater prevalence in children below 3 y of age, those with convulsions or coma, hyperparasitaemia, and those receiving quinine treatment [31]. The pathophysiology is multifactorial and increased glucose utilization, impaired gluconeogenesis, and hyperinsulinemia secondary to quinine therapy all contribute [27,32–34]. Many studies have demonstrated that hypoglycemia is an important risk factor for death (Table 1)[22,26,27,35,36,237].

### Metabolic acidosis

Metabolic acidosis (Table 1) is a common finding (43–46% prevalence) in studies of African children admitted to hospital with malaria [31,37,38]. It is thought to occur due to a combination of microvascular obstruction by sequestered parasites, vascular endothelial dysfunction, anemia, and inflammation driving increased glycolysis. Metabolic acidosis is strongly associated with an increased risk of death (Table 1)[16,35,36,39]. In the AQUAMAT trial, the severity of acidosis was associated with increasing mortality (pH 7.2 associated with almost 20% mortality; pH <7.0 associated with >60% mortality [14]). Hyperlactatemia is a major contributor to the acidaemic state in malaria, along with reduced plasma bicarbonate. Prognosis has been shown to be worse in patients whose lactate levels do not normalize within a few hours of admission and the levels of lactate in fatal cases were nearly double those of survivors (7.1 mmol/L in fatal vs. 3.6 mmol/L in survivors) amongst Gambian children [40].

### Respiratory distress

Respiratory distress (Table 1) describes abnormal, deep and effortful breathing (chest-indrawing or nasal flaring) with an increased respiratory rate for age. The incidence of respiratory distress in SM ranges from 7-41% [10,31,41] and it is a major risk factor for death, but with highly variable estimates of effect size (Table 1).It is present in over half of reported malarial deaths [10,14,22,36,42]. In childhood malaria, respiratory distress usually reflects underlying metabolic acidosis while pulmonary edema (Table 1) and associated acute respiratory distress syndrome are rare in children but more commonly seen in adults [43]. Respiratory distress and metabolic acidosis as a clinical syndrome and markers of prognosis are considered in further detail below.

### Renal failure

Renal dysfunction associated with malaria is due to acute tubular necrosis and less commonly interstitial nephritis and glomerulonephritis; multiple mechanisms including parasitized erythrocytes causing tissue hypoperfusion and immune complex deposition have been proposed [44,45]. The optimal assessment of renal function requires serial biochemical measurements of blood and/or urine using a patient’s baseline renal function as a reference [46]. Unfortunately, this is not routinely available in most malaria endemic settings. As diagnostic facilities have improved in some settings, it has been recognized that the burden of acute renal impairment in malaria is substantial [45,47]. A recent Ugandan study assessing the renal function of children with SMA and CM reported a prevalence of over 35% [48]. Results of many clinical studies investigating SM have supported the prognostic significance of renal dysfunction (Table 1). Additionally, von Seildlein et al. showed a statistically significant, but small, rise in mortality with increased blood urea nitrogen in a multivariable predictive model (blood urea nitrogen >20 mg/dL AOR 1.02 [95% CI 1.02–1.03])[14]. Moreover, the presence of acute renal impairment has not only been shown to be a risk factor for the development of chronic kidney disease (OR 2.81 [95% CI 1.02–7.73]) but also for long-term neurological impairment in CM (AOR 3.03 [95% CI 1.22–7.58]) at 2-y follow-up [48].

### Thrombocytopenia

Thrombocytopenia is defined as platelet levels lower than expected for age. While it is a well-documented finding in SM [49], it rarely causes symptoms. Several underlying mechanisms have been postulated including immune-mediated destruction, splenic sequestration, and bone marrow suppression [49]. Literature on the prognostic value of thrombocytopenia in malaria is contradictory and relatively limited (Table 1). Several studies have reported its usefulness in aiding malaria diagnosis (platelets <150, sensitivity 60%, specificity 88% [50]) and as a predictor of mortality (Table 1) but have varying results due to their different definitions of thrombocytopenia. Furthermore, other studies found that while platelet levels were lower in SM compared to UM patients, this did not have any association with outcome [51,52].

### Bleeding

Overt bleeding associated with malaria is uncommon and can be secondary to multiple pathological processes including coagulopathy and thrombocytopenia [53], which can occasionally manifest as bleeding from the mucous membranes and venepuncture sites [17]. When symptoms are present, it is indicative of a more complicated disease process and hence could be speculated to be associated with greater mortality. However, due to its rarity, no studies have formally assessed its value as a marker of prognosis (Table 1) and the majority of the literature is from case series and reports of adult malaria [54–57].

### Severe malaria anemia

SMA is the most common complication of *P. falciparum* infections, affecting the youngest children in areas of high transmission [58–62], manifesting clinically with pallor, lethargy, and poor feeding. The WHO define SMA as hemoglobin <5 g/dl or a hematocrit of <15% in children (aged <12 y) with a parasite count >10,000/microliter [17]. SMA is a major cause of hospitalization [60,63] and is associated with poor prognosis and increased mortality, although estimates of effect size vary (Table 1) [22]. Substantial work has been carried out to understand the pathophysiology of SMA and its value as a prognostic indicator which will be discussed in further detail below.

### Prostration

Prostration is a sign of weakness and may also include mild cerebral impairment, resulting in the inability to sit unassisted or to breastfeed in those under the age of 6 months [14,17]. It can progress to a coma or be present in the post-ictal phase following a seizure. While prostration is commonly reported in SM [10,14,64], it can be a subjective observation [17]. This is likely one of the reasons why it has variable prognostic value in studies in African children (Table 1) [65] ranging from small to moderate increase in the risk of death, and usually considerably less increase in risk than in children with other features of SM (Table 1) [14,22,36]. This is consistent with the WHO assessment that prostration has a low prognostic value (Table 1).

### Neurological abnormalities

The neurological abnormalities in SM include seizures and impaired consciousness (Table 1). The severity of impaired consciousness is often measured using the Blantyre coma scale. This is a modified version of the Pediatric Glasgow Coma Scale, specifically constructed to allow rapid and straightforward assessment of the severity of malaria-induced coma. Impaired consciousness and seizures are associated with a higher risk of mortality in pediatric malaria patients (Table 1)[16,22,36,37,66]. It is important to note that the definition of coma varies between studies and that some studies do not state a formal definition (Table 1). This has resulted in some variability in the effect size estimation of impaired consciousness (Table 1). The clinical syndrome CM, which encompasses these neurological symptoms is considered in further detail below.

### Jaundice

Jaundice is yellowing of the skin, mucosal surfaces, and whites of eyes caused by excess bilirubin, which can be detected by clinical examination. As a feature of SM, it is defined as raised plasma bilirubin (>50 µM or 3 mg/dl) with evidence of significant parasitemia (>100,000 µl) [17]. It is caused by a combination of hemolysis and hepatic dysfunction and is more common in adults, though it can also be a presenting feature in neonatal malaria [17,66]. Several studies have identified the presence of jaundice as a risk factor for mortality in children with malaria in univariable analysis (Table 1). In one large study, the mortality in cases with jaundice was 22/114 (19%), whilst without jaundice 505/5312 (10%) (p < 0.001) [14]. However, multivariable analyses have not consistently identified jaundice as an independent predictor of mortality when combined with other features of SM (RR 2.6 [95% CI 1.1–6.3] [10]; OR 0.87 [95% CI 0.31–2.49] [23]), possibly because it is relatively uncommon compared to some of the other features of SM.

### Shock

Shock (Table 1) is a life-threatening condition caused by circulatory failure leading to tissue hypoxia. If recognized and treated in its early stages (compensated shock), it can be reversible, however, it can progress quickly to multiorgan failure and death [67]. In recent years, two large multicentre studies have analyzed the risk of death following shock in malaria. von Seidlein et al. showed a significantly increased mortality rate of 19% in children with shock vs. 9% in those without, in a cohort of children with SM [14]. In another study, the presence of compensated shock was associated with an increased risk of death (Table 1) on univariable analysis but not in the multivariable analysis due to the effect of shock on mortality rate being accounted for by the other covariates included into the models [23]. Table 1 illustrates the fairly consistent moderate estimates of the effect of this feature on mortality in univariable analysis. This is reflected by the WHO giving this feature a high prognostic value.

### Independence of prognostic markers

Due to the complex, interlinked pathophysiology, many of the features of SM occur together and are correlated. Researchers have tried to identify features with the greatest independent prognostic value using multivariable models.

The most commonly recognized independent factors include metabolic acidosis [14,37,40,68–71], hypoglycemia [10,29,40,68–70], impaired consciousness [10,14,29,64,68–71], renal dysfunction [14,29,69] and respiratory distress [10,29,64,69–71]. Those less frequently described in the literature include: the presence of an underlying chronic illness [14], SMA [68,71] and parasite stage visible by microscopy [69]. Combinations of these scores allowed for the prediction of the majority of malaria deaths, for example 92% of the deaths in a cohort of 1844 children (average age: 26.4 months) with a mortality rate of 3.5% were predicted by a model incorporating four features: respiratory distress (RR 3.9 [95% CI 2.0–7.7]), impaired consciousness (RR 3.3 [95% CI 1.6–7.0]), hypoglycemia (RR 3.3 [95% CI 1.6–6.7]), and jaundice (RR 2.6 [95% CI 1.1–6.3])[10]. von Seidlein et al. also combined several clinical scores into a multivariable model of 13 clinical features, which had an area under the curve (AUC) of 0.85 [95% CI 83–87] [14].

Recent metanalyses have also helped to elucidate the strength of multiple prognostic indicators in predicting mortality in SM. Sypniewska et al. evaluated 36 variables from 30 studies of children in Africa with SM; variables most strongly associated with death included renal failure (OR 5.96 [95% CI 2.93–12.11]), coma (OR 4.83 [95% CI 3.11–7.5]), hypoglycemia (OR 4.59 [95% CI 2.68–7.89]), shock (OR 4.31 [95% CI 2.15–8.64]), and deep breathing (OR 3.8 [95% CI 3.29–4.39]) [72]. Leopold et al. applied a causal inference model to a pooled data set of adults and children with SM from 15 countries across Asia and Africa. They reported similar findings of increased mortality associated with acidosis (OR 2.10 for a 7 mEq/L increase in base deficit [95% CI 1.93–2.28]), renal impairment (OR 1.71 for a 2-fold increase in blood urea nitrogen [95% CI 1.58–1.86]), coma (OR 3.59 [95% CI 3.07–4.21]), seizures (OR 1.40 [95% CI 1.16–1.68]), shock (OR 1.51 [95% CI 1.14–1.99]), and presumed pulmonary edema (OR 1.58 [95% CI 1.04–2.39]) [73].

Score systems have been developed to simplify the application of prediction models for rapid implementation in clinical settings. For instance, Helbok et al. developed the Lambaréné Organ Dysfunction Score (LODS) 0–3, which denote the presence of coma, prostration, and deep breathing – the three variables which were most significantly associated with fatal outcomes in six sites across Africa from the SMAC network [64]. The LODS was found to be a simple and useful predictor of mortality (OR for a 1-unit increase in the LODS was 3.53 [95% CI 3.30–3.77])[64]. A second example is Pediatric Early Death Index for Africa (PEDIA) which uses multiple clinical variables with different weighting (jaundice (weight, 1.0); subcostal indrawing (weight, 1.0); prostration ± seizure (weight, 2.0); altered consciousness (with seizures, weight, 2.0; without seizures, weight, 3.0); wasting (weight, 1.0)) [74,75]. PEDIA effectively discriminated survivors and non-survivors in a prospective subgroup analysis performed by Conroy et al. with AUC 0.92 [95% CI 0.90–0.93] [75].

## Prognostic biomarkers of severe malaria

In addition to clinical features, many studies have also identified molecular biomarkers associated with adverse outcome in children with malaria. Whilst SM is a heterogeneous condition defined by the variety of clinical manifestations outlined above, biomarkers that predict progression from UM to SM and outcome across all SM phenotypes would be extremely attractive in clinical practice. Potential prognostic biomarkers originating from both host and parasite have been described.

### Parasite biomarkers

Plasma levels of *P. falciparum* Histidine Rich Protein-2 (PfHRP2) have been demonstrated in multiple studies to be associated with the severity of malaria in children [11,76]. PfHRP2 is a parasite protein which is predominantly released from infected blood cells at the time of schizont rupture, and has a relatively long half-life, meaning that levels accumulate in plasma as parasite load increases [77]. PfHRP2 is widely believed to be a better indicator of total body parasite load than parasitemia, because plasma levels of the protein should represent both sequestered and circulating parasites. PfHRP2 concentrations have consistently been found to increase as the severity of infection increases, characterized by the presence of more severe symptoms including coma, acidosis, SMA, and death [11,76,78,79]. For example, in a large study (n = 3,826) Hendriksen et al. found that the mortality of SM patients increased by about 20% for every log_10_ increase in serum concentration of PfHRP2 above 174 ng/mL [79].

Furthermore, higher plasma concentrations of PfHRP2 are associated with the presence of the CM phenotype (CM: n = 25, UM: n = 125; CM mean PfHRP2 concentration: 7838 ng/mL [SD, 2.5 ng/mL], UM mean PfHRP2 concentration: 421 ng/mL [SD, 9.0 ng/mL], p-value < 0.001 [80]). PfHRP2 is also important because it is one of the antigens detected in many rapid diagnostic tests for *P. falciparum* [81]. The potential utility of quantitative PfHRP2 tests for prognosis is still being explored. One challenge for this approach is that the concentrations of PfHRP2 associated with SM seem to differ greatly between different transmission settings (in a similar way to the relationship between parasitemia and severity) and so establishing universal thresholds for outcome prediction is unlikely to be possible.

Hemozoin, the by-product of the digestion of hemoglobin by *Plasmodium* parasites, could be an indirect marker of parasite load. Its presence in parasitized erythrocytes, neutrophils and to a lesser extent monocytes, was associated with increased mortality risk at most sites in the SMAC cohort [36]. However, the C-statistics (equivalent to the AUROC) for these markers ranged from 0.50 (erythrocytes) to 0.70 (granulocytes) [36] indicating relatively low prognostic value. This might be explained by the fact that hemozoin cannot be degraded by phagocytic cells, so its accumulation may be more reflective of cumulative than current parasite load.

Another parasite molecule strongly associated with the outcome of infection is the *P. falciparum* erythrocyte membrane protein 1 (PfEMP1). PfEMP1 proteins are parasite antigens encoded by *var* genes and transported onto the surface of parasitized erythrocytes [82–84]. They bind cell surface receptors such as CD36 on the vascular endothelium to facilitate the sequestration of parasitized red cells within small blood vessels and avoid transit through the spleen [85]. Only one *var* gene is expressed by an individual parasite at any time; however, in each new generation, daughter parasites can switch expression amongst the 60+ *var* genes in their genome. The *var* genes and the PfEMP1 proteins they encode are highly polymorphic, enabling them to evade host antibody responses [86,87,]. Despite this, it has been possible to classify them into groups with conserved structural and biological properties.

Preferential expression of different subsets of PfEMP1 is associated with different disease manifestations [88–93]. Having a significantly higher proportion of *var* genes that are group A and/or B that contain domains that bind the Endothelial Protein C Receptor (EPCR) has consistently been found to be associated with the SM phenotypes CM and SMA in different populations (mixed adult and child cohorts [91–94]). It is thought that PfEMP1 binding to EPCR not only facilitates sequestration and increased parasite load, but also has direct pathogenic effects by impeding the function of EPCR and leading to dysregulation of vascular inflammation, coagulation, and the permeability of the vascular endothelium [95]. Indeed, a recent study has shown that the combination of *var* expression profile and parasite load is a better predictor of clinical phenotype (SM vs. UM) than parasite load alone [96], however, the study was not designed to detect association with survival. Although the determination of *var* gene or PfEMP1 expression might have useful prognostic value, applying this to routine clinical practice would currently be challenging due to the extensive genetic variability and complexity of classification of *var* genes.

Parasite molecules may be associated with prognosis, independent of any role in pathogenesis, if they influence the success of antimalarial treatment. Numerous genetic variants causing or associated with resistance to different antimalarials have been identified in *P. falciparum* [97,98], including emerging resistance to current first-line artemisinin-based combination therapies [99]. Available evidence indicates that being infected with parasites resistant to the empirical antimalarial treatment is likely to result in an increased risk of adverse outcome, although data quantifying this risk for specific combinations of resistance mutations and antimalarial drugs is not widely available [97,100]. Nevertheless, rapid identification of resistance mutations at the point-of-care could help to ensure that individuals receive optimal treatment.

### Host biomarkers

An appropriate host inflammatory and immune response to infection is vital to help clear parasites from the circulation, whilst an inappropriate or excessive response could result in damage to host tissues. Thus, there has been great interest in the potential of components of the host inflammatory and regulatory responses as predictors of outcome. For example, the prototypical pro-inflammatory cytokine, tumor necrosis factor-α (TNF), was one of the first to be found to be expressed at higher levels in SM than UM, and at highest levels in fatal cases, and this has been confirmed repeatedly since [101–103]. These findings led to adjunctive clinical trials of anti-TNF monoclonal antibody treatment in SM, which ultimately showed no improvements in outcome [103].

Interleukin 10 (IL-10) is an anti–inflammatory cytokine also implicated in the dysregulation of inflammation. In mouse models, it has been found to be protective against SM phenotypes, including CM [104–106], but has also been found to reduce the ability of the host to clear the parasites from the blood [107]. Studies of African children indicate that IL-10 may prevent the manifestation of some severe phenotypes by suppressing the expression of TNF [108] but in doing so may promote the growth of the parasite, which in itself can cause SM [109,110].

The ratio of different combinations of inflammatory and regulatory cytokines has also been highlighted as important in balancing the inflammatory response. For example, higher ratios of transforming growth factor-beta (TGF-β1)/IL-12 and IL-10/IL-12 have been found in children with SM relative to UM [102].

Differences in the levels of numerous other plasma proteins and small molecules have been observed in comparisons of children with SM with UM, and some of these have been evaluated as prognostic markers. For example, the soluble form of triggering receptor expressed on myeloid cells-1 (sTREM-1), whose function is to amplify the inflammatory response of neutrophils and monocytes, and soluble vascular endothelial growth factor receptor 1 (sFlt-1), which is involved in cell proliferation, has been associated with outcome. With plasma concentration cutoff points of >289.9 pg/mL and >1066.3 pg/mL for sTREM-1 and sFLT-1, respectively, both achieved high sensitivity values of 95.7% [95% CI 78.1–99.9] and 82.6 [95% CI 61.2–95.0], respectively, for predicting mortality in SM patients, but specificity was low (sTREM-1: 43.8% [95% CI 32.7–55.3], sFlt-1: 57.5 [95% CI 45.9–68.5]) [111].

In contrast, the cytokine interferon gamma-induced protein-10 (IP-10), which is involved in the chemoattraction of T cells, natural killer cells, and monocytes, achieved high values for both sensitivity of 82.6% [95% CI 61.2–95.0] and specificity of 85% [95% CI 75.3–92.0] for mortality prediction in this cohort [111] when the plasma concentration was >831.2 pg/mL. Other inflammatory response proteins associated with mortality in SM include high-mobility group protein-1 (HMGB1, a cytokine) [112], monocyte CD36 expression [113] and von Willebrand factor (VWF, a glycoprotein in the blood) [114]. Thus, many proteins associated with inflammation have potential prognostic value in SM, however, many require further validation in different patient cohorts to better assess this potential.

It is likely that the combination of multiple host biomarkers will be needed to produce widely applicable predictive tests for the outcome of malaria. The selection of optimal panels of biomarkers remains to be achieved, although modern variable selection and machine learning approaches should make this easier [115,116]. The incremental benefit of biomarkers above the predictive value of clinical features should be considered, as well as the possibility of prediction rules combining both clinical features and biomarkers to achieve the best possible prediction.

## Clinical syndromes and specific prognostic biomarkers

In addition to prognostic features and biomarkers for SM in general, there are predictors of outcome specific to each of the major phenotypes of SM which occur in children: CM, SMA, and respiratory distress. Since there are clear clinical and biological differences between these syndromes [11], a more tailored approach to prognosis may improve prediction for individual patients.

### Cerebral malaria

#### Mortality

CM is one of the most feared clinical complications of malaria. The pathogenesis is still debated, but in *P. falciparum* malaria the sequestration of parasites in the microvasculature of the brain appears to be a necessary feature, and cerebral edema (brain swelling) appears to be the final common pathway leading to death. Mortality rate is variable depending on the setting and presence of other SM features, however, even when these are accounted for it can still be as high as 15-25% in tertiary care centers [117,118].

CM is diagnosed in a patient with *P. falciparum* identified on a peripheral blood smear, with one or more neurological symptoms including reduced consciousness, coma (Blantyre coma score ≤ 2) and seizures, where other potential causes have been ruled out. Other causes could include hypoglycemia, hyperpyrexia, acidosis, SMA, medication, post-ictal phase, as well as encephalopathy caused by a co-infection. How to exclude these other causes and thus identify the true-CM patients has been discussed at length [37,119,120].

In 2011, Beare et al. suggested that the presence of malaria-specific retinopathy (changes at the back of the eye which can be seen during clinical examination) should be required for the diagnosis of CM [121]. These features include retinal whitening, hemorrhages, and changes to the vasculature. The vasculature of the retina and brain has a common embryonic origin, and so changes in the retinal vessels are thought to be representative of the sequestration occurring in the brain microvasculature [122]. The severity of retinopathy is also being investigated as an independent predictor of mortality in CM patients [123]. In one study the OR for mortality based on the presence of any signs of retinopathy was 5.5 [95% CI 1.3, 23.4] [123]. Whether retinopathy-negative patients with features of CM and no alternative diagnosis genuinely have CM remains a subject of debate.

A substantial amount of work has been conducted to identify what distinguishes fatal-CM from non-fatal-CM. Sequential studies using increasingly powerful Magnetic Resonance Imaging (MRI) scanners have revealed that increased intracranial pressure due to cerebral edema is the cause of death in African children with CM [118,124–126]. Higher-resolution scans have been used to try and understand the etiology of the cerebral edema and have identified obstruction of the cerebral microvasculature, microhaemorrhages, and evidence of autoregulatory dysfunction [127]. A small study of Indian children with CM indicated that impairment of the blood-brain barrier (due to sequestration) was responsible for the swelling in the posterior region of the brain [128]. However, another small study in Taiwanese children with UM also found some similar MRI abnormalities in the absence of CM [129]. The prognostic value of specific MRI features remains to be defined and at present, the potential of MRI for routine risk stratification is unrealistic in most malaria endemic settings due to the cost and logistic challenges of scanning seriously ill children.

Electroencephalography (EEG), which measures the electrical activity of the brain, has also been used to try to identify predictive features for mortality in CM. Large studies of children with CM in Malawi and Uganda found that lower average voltage, lower maximum voltage, and a lack of reactivity were all associated with death [118,130]. However, further research is required to better understand the added value of EEG as a prognostic tool.

Many potential molecular biomarkers have been identified as associated with CM mortality. Biomarkers have been identified at the proteomic level (i.e. 266 host and parasite proteins identified by Moussa et al. [131]) and at the genetic and metabolomic levels (i.e. 19 plasma metabolites identified by Gupta et al. [132]) and originate from both the host and parasite. Those from the host are mainly involved in inflammatory responses (Table 2). Many of these biomarkers require prospective validation in larger cohorts of patients before their clinical utility can be accurately assessed.

**Table 2.** Examples of proteins and genes identified as associated with CM mortality.

Molecule(s)	Role(s)	Sample Size	Location	Age Range	Main Findings
**Chitinase-3-like protein 1**	Secreted glycoprotein expressed in inflammation and fibrosis	Fatal-CM (n = 23), Non-fatal CM (n = 80)	Uganda	6 months to 12 y	Elevated plasma chitinase-3-like protein 1 expression was predictive of mortality in CM patients (AUC: 0.84 [95% CI 0.76–0.92]) [238].
**Angiopoietin-2**	Vascular growth factor	CM (n = 155)	Malawi	6 months to 14 y	Elevated expression in fatal-CM (AOR 3.9 [95% CI 1.2–12.7], cut-point: > 3.85 ng/mL) [239].
**Tie-2**	Angiopoietin receptor	CM (n = 155)	Malawi	6 months to 14 y	Elevated expression in fatal-CM (OR 3.2 [95% CI 1.6–6.3], cut-point: > 67.8 ng/mL) [239].
**Angiopoietin-1**	Vascular growth factor	CM (n = 155)	Malawi	6 months to 14 y	Low expression associated with fatal-CM (OR 2.5 [95% CI 1.0–5.9]). Levels ≤ 6.76ng/mL were also associated with the presence of retinopathy (OR 5.9 [95% CI 2.7–12.8]) [239].
		UM (n = 67), CM (n = 69), Healthy (n = 28)	Uganda	4 to 12 y	Low serum expression levels were associated with death in pediatric CM patients (median level in non-fatal CM: 9.1, range: 0.39–38; fatal CM: 0.39, range: 0.39–4.6; p -value: 0.027) [240].
**L-arginine**	Protein biosynthesis and nitric oxide synthesis	CM (n = 39), Healthy (n = 19), UM (n = 17)	Tanzania	1 to 8 y	Plasma levels < 45.4 µmol/L were associated with fatal-CM (OR 26.2, [95% CI 1.2–586] in multivariate analysis) [241].
**TNF-alpha2 allele (**rs1800629)	Pro-inflammatory cytokine	Fatal-CM and sequelae (n = 99), non-fatal and no sequelae (n = 277)	Gambia	1 to 10 y	Identified a RR of 7.7 for mortality in CM patients homozygous for this allele ([95% CI 1.9–30.0]) [198].
**Erythropoietin**	Hematopoietic Factor	CM with plasma erythropoietin measured (n = 204)	Uganda	18 months to 12 y	High plasma levels associated with more severe CM as characterized by longer coma and a 1.74-fold [95% CI 1.09–2.8] increase in mortality[156].

**Legend**

Abbreviations: Area Under the Curve (AUC); Adjusted Odds Ratio (AOR); Confidence Interval (CI); Cerebral Malaria (CM); Odds Ratio (OR); Relative Risk (RR); Uncomplicated Malaria (UM)

The vascular growth factors, angiopoietin-1, and angiopoietin-2, involved in angiogenesis and regulation of vascular endothelial function, are two of the most well-established biomarkers for prediction of outcome and there is evidence from experimental CM in mice of the former having a mechanistic role in the development of CM [112]. These proteins are predictors of mortality for both CM specifically as well as the general SM phenotype in a range of settings and populations, with angiopoietin-2 achieving area under the receiver operating characteristics (AUROC) values between 0.71 and 0.83 (Table 2) [111,133,134]. These proteins are also currently being investigated as therapeutic targets [134].

#### Neurological sequelae

Survivors of CM are at risk of long-term neurological impairments (neurological sequelae) including deficits in cognitive, motor, and/or sensory function. The most common neurological sequelae following CM in children are muscular weakness, co-ordination/balance, and speech problems [135,136]. The attribution of such broadly defined neurological sequelae to malaria is challenging [137], even with expensive longitudinal studies [136]. However, the best estimates indicate that 5-25% of CM survivors will exhibit some form of neurological sequelae 6 months after discharge depending on how carefully they are assessed [135,138–140]. Additionally, around 10% of pediatric CM survivors are thought to develop epilepsy [139,141].

The most well-established predictors of neurological sequelae appear to be the depth and duration of coma [29,136,142] and the presence of convulsions [135,143–145]. However, Jallow et al. found that having a Blantyre coma score of equal to or less than 2 predicted the development of neurological sequelae with only modest accuracy (sensitivity: 0.74, specificity: 0.69, AUC: 0.72, [95% CI 0.67–0.76]) [29]. Many of these features also share molecular biomarkers with neurological sequelae [146–149], such as kynurenine, which is associated with the duration of coma as well as numerous measures of neurological sequelae including attention deficit [147]. The severity of retinopathy has also been linked to the presence of neurological sequelae, with papilledema, disk hyperemia, macula whitening, and foveal annulus being most strongly associated with neurodevelopmental test outcomes [150].

Neurological sequelae have also been reported in children with SM and UM who did not have CM, when measured relative to healthy controls [151]. For example, Ugandan patients who had SMA had more behavioral problems relative to age matched healthy controls at 12 and 24 months after their index malaria episode [136]. Despite adjusting for potential confounders in the analysis, it is unclear whether this is a direct effect of malaria (i.e. brain damage) or an indirect effect (for example, due to preexisting poor nutrition or reduced learning opportunities).

The relationship between hypoglycemia and neurological sequelae is also uncertain. A case-control study in Nigerian children identified that hypoglycemia was more common in children with neurological sequelae at discharge after CM (7/11 children, 63.6%) than those without neurological sequelae (7/51 children, 13.7% [152]). However, a subsequent larger study of 160 children (13.7% of survivors had neurological sequalae on discharge) in the same setting found no statistically significant association between hypoglycemia and the presence of neurological sequelae [140] and nor did a large study of 624 children (23.3% with neurological sequalae on discharge) conducted in the Gambia [135]. One explanation for these discrepancies may be due to the latter two studies having more representative cohorts of patients with neurological sequelae, or due to differences in the time to recognition and treatment of hypoglycemia between these studies.

Other molecular biomarkers have also been identified as predictors of neurological sequelae in pediatric malaria, most notably, erythropoietin [145]. Erythropoietin, a hormone and cytokine best known for controlling the production of red blood cells, has been identified as a potential protectant against neurological damage in a variety of settings [153]. Plasma levels of erythropoietin greater than 200 units per liter have been found to be associated with an 80% reduction in the risk of long-term neurological impairment in CM [145]. However, evidence from its use in clinical trials to treat other neurological conditions such as cerebral ischemia (against which it has also been shown to be neuroprotective [154]) has suggested that high erythropoietin levels can also result in higher fatality rates [155]. This is consistent with another study which found that high plasma levels of erythropoietin were associated with longer coma in CM and a 1.7-fold increase in mortality [95% CI 1.09–2.8] [156], but only in children with Hb levels <8 g/dL. This further complicates the potential utility of erythropoietin as a prognostic biomarker for CM patients.

Vascular endothelial growth factor (VEGF) was also investigated by Casals-Pascual et al., but found not to be neuroprotective [145]. However, its plasma levels were inversely correlated with erythropoietin and it was associated with the presence of seizures (OR: 4.1 [95% CI 1.75–9.60]) [145].

Proteins and metabolites in the cerebrospinal fluid (CSF) have been identified as potential biomarkers of neurological damage and predictors of neurological sequelae in CM. For example, high CSF (but not plasma levels) [157] of TNF were significantly associated with the occurrence of neurological impairment in one study of Ugandan children, but with a relatively small effect size and only in children older than 5 y [148]. Tau protein levels in CSF were associated with a greater severity of neurological sequelae assessed by overall cognitive scores, working memory, and attention at 2 y after discharge [149]. CSF tau protein levels measured at hospital admission predicted the presence of neurological sequelae at time of discharge relatively poorly (AUC 0.62 [95% CI 0.52–0.72]), but were a better predictor of persistent deficits at 24 months (AUC 0.89 [95% CI 0.8–0.99] [149]), suggesting that this could be a promising biomarker. Elevated CSF kynurenic acid and kynurenine at presentation have also been found to correlate with decreased scores in behavioral tests at 12 months after index CM episode [147,148]. However, all these biomarkers require validation in different pediatric patient cohorts, and many seem to only be associated with specific aspects of cognition in older children (>5 y).

### Severe malarial anemia

SMA (Table 1) is one of the most common complications associated with childhood SM in endemic settings, resulting in a high burden for health services [158]. SMA can arise chronically from repeated or persistent infections or acutely due to severe hemolysis, sometimes associated with blackwater fever. Blackwater fever is a rare complication in children characterized by rapid intravascular hemolysis, fever, and acute renal failure; reported in association with quinine use and glucose-6-phosphate dehydrogenase deficiency [159–161] and associated with increased risk of mortality (HR 3.37 [95% CI 1.3–8.5]) and hospital readmission (HR 1.68 [95% CI 1.1–2.5]) [162]. Hemolysis has also been reported to occur 1–2 weeks following treatment with artesunate, primarily in nonimmune travelers, resulting in an increased need for blood transfusions [163].

Multiple factors can contribute to SMA, including co-infections with HIV, helminths (such as hookworm), and bacteria, and nutritional deficiencies [62,164,165]. Furthermore, malaria-driven natural selection for hemoglobinopathies and erythrocyte abnormalities themselves result in increased risk of severe anemia during an acute malarial illness [166–170]. Alongside hemolysis of parasitized erythrocytes, there is accelerated destruction of non-parasitized erythrocytes, becoming more prominent with increasing disease severity [158,171–174]. Abnormal erythrocytes can be sequestered by the rapidly enlarging spleen, sometimes progressing to massive hyper-reactive splenomegaly [175]. There is additional suppression and dysregulation of erythropoiesis during the acute illness [176–178] thought to be triggered by the host immune response and associated with intramedullary deposition of malaria pigment, hemozoin [179–181]. The disease process is further exacerbated by slower reticulocyte response in immunologically naive individuals and repeated infections restricting bone marrow recovery [36,158].

Risk of death in SMA is dependent on several factors including the degree of anemia [14,182], rate of disease progression [17,183], age, transmission intensity [184], access to and quality of health services, and availability of prompt transfusions [158]. The relationship between these factors and SMA is consistently stated in the literature but the effect is not well quantified. If SMA is accompanied by another clinical syndrome, particularly respiratory distress, the mortality rates are much higher (SMA alone ~1%; SMA and severe respiratory distress ~16% [10,185]) but outcomes can be improved with timely blood transfusions [42,186].

There is also evidence from clinical studies, epidemiological findings, and mouse-models for proposing a causal relationship between SMA and risk of bacterial co-infection, and hence higher case fatality [187,188]. The most plausible mechanism being hemolysis resulting in favorable conditions for bacterial growth and impaired neutrophil function [188].

Using a causal modeling approach, Leopold et al., showed that moderate anemia (hematocrit 15–25%) was in fact protective against mortality (OR 0.87 for a decrease of 10 percentage points in hematocrit [95% CI 0.80–0.95]) with no evidence for a beneficial effect of transfusion (OR for death in the transfused group of 0.99 [95% CI 0.97–1.02]) [73]. This protective effect was not seen in the retrospective analysis by Maitland et al., where reduction in admission hemoglobin levels was associated with a small increase in odds of mortality (hemoglobin 6–6.9 OR 1.03[95% CI 0.95–1.12]; hemoglobin 5–5.9 OR 1.18 [95% CI 1.08–1.30]; hemoglobin 4–4.9 OR 1.12 [95% CI 0.97–1.29]; hemoglobin 3.9–3 OR 1.33 [95% CI 1.06–1.69]) with greatest mortality risk at hemoglobin levels below 3 g/dl (OR 6.36 [95% CI 4.21–9.62]) [189].

Treatment with blood transfusion is recommended when hemoglobin levels fall below 5g/dl [18]. While lifesaving, especially in settings where readily and safely available [31], transfusions can carry a significant risk of transmitting other bloodborne infections such as HIV [190]. In the past, up to 50% of children admitted with malaria in some hyperendemic settings received blood transfusions [42,191,192]. Retrospective analysis in Kenyan children suggests that pre-transfusion hemoglobin levels may influence the potential benefit of blood transfusion. Perhaps surprisingly, transfusion at a baseline hemoglobin level of 4–4.9 g/dL was associated with increased risk of death (OR of death 3.29 [95% CI 1.21–8.94], compared to un-transfused children), while mortality tended to be lower in transfused children with pre-transfusion hemoglobin less than 3.9 (Hb 3.9–3 g/dL, OR 0.59 [95% CI 0.21–1.69]; Hb < 3g/dL OR 0.72 [95% CI 0.29–1.78]) though these values were not statistically significant, possibly due to lack of statistical power [189].

Many biomarkers associated with the presence of/or susceptibility to SMA have been identified. Interesting findings include a dysregulated immune response, primarily Th1 cytokines, and chemokines, and their association with dyserythropoiesis [193–197]. Genetic variants that confer either susceptibility (TNF promoter variants [198]; IL-10 promoter variant [199]) or protection against (SCFC promoter variant [200]) SMA have also been identified. While this work has furthered our understanding of underlying pathophysiological mechanisms, the roles of these biomarkers as prognostic indicators in the clinical setting are limited as clinical symptoms and basic laboratory tests adequately identify the clinical syndrome and its severity. Further work is required to identify biomarkers and signatures of poor prognosis following the development of SMA and indicators of associated long-term complications.

### Respiratory distress and metabolic acidosis

In childhood malaria, respiratory distress is largely attributed to metabolic acidosis (Table 1) and is less commonly due to parenchymal lung disease or heart failure related to anemia [17,201,202]. Respiratory distress due to malaria shares many clinical features with pneumonia, and it is often difficult to distinguish the two when diagnostic facilities are limited [203–205].

Over 80% of children with SM complicated by respiratory distress are found to be profoundly acidotic [10]. Moreover, deep breathing can accurately predict the presence of acidosis (sensitivity 91%, specificity 83% [202]). When appropriate tests are available, metabolic acidosis has consistently been identified as an independent predictor of mortality [10,14,37,40,69,206,207]. In a meta-analysis of studies the OR for death was 2.3 [95% CI 1.2–4.4] [72]. Furthermore, high (78%) and early mortality was reported in cases with hyperkalemia in the context of acidosis in SM [208].

The principle cause of acidosis is thought to be tissue hypoxia and resultant anaerobic glycolysis generating lactic acid, and additional accumulation and impairment of the metabolism of lactic acid [40] and ketone bodies [38,209]. There is emerging evidence for the role of other organic acids, including hydroxyphenyllactic acid (HPLA), α-hydroxybutyric acid, and β-hydroxybutyric acid [210] and numerous products of the gut microbiota discovered in a recent metabolomic study [211]. Further research is necessary to define if levels of these more recently identified metabolic acids will improve the prediction of outcome.

## Coinfections and prognosis

### Bacterial coinfection

Malaria, young age, malnutrition, and HIV infection were identified as the strongest risk factors for bacteremia amongst children admitted to hospital in a large study in Kenya [212]. Children with SM are known to have a higher prevalence of bacteremia; most commonly non-typhoidal Salmonella [212–214]. Several pathological processes have been proposed including translocation of gram-negative organisms across the bowel wall, specific macrophage and neutrophil dysfunction, and functional hyposplenism [187 188 215,,]. Bacterial co-infection in malaria patients has been associated with a higher risk of death in some [212 216,] but not all studies [217].

### HIV coinfection

The geographical overlap between high malaria incidence and HIV prevalence presents a substantial burden caused by coinfection. Whilst literature on the pediatric population is limited, due to the lower prevalence of HIV in children, studies have shown that HIV-infected individuals of all ages are more susceptible to SM [218–224] – especially in areas of unstable endemicity [17,223,225]. Co-infected individuals have increased parasitemia, a more complicated disease course, higher morbidity, and higher mortality. For example, one study [219,226] identified a mortality of 26% (19/74) in HIV-coinfected children versus 9% (53/581) in uninfected children. Increased mortality rates have been observed in HIV co-infected children with CM compared with HIV-uninfected children with CM (OR 1.4 [95% CI, 1.1–1.9][227]). Co-infected children are also at greater risk of severe anemia (OR 8.71 [95% CI 3.37–22.51])[218,228].

## Prognosis of malaria in nonimmune travelers

Travelers to malaria endemic areas may be malaria naïve with no naturally acquired immunity or may have waning immunity even if they were previously resident in the endemic country. Most life-threatening malaria complications occur in nonimmune travelers returning from malaria endemic areas, with 5% of those affected suffering SM [229]. The mortality rate in malaria cases in Europe was 0.4%-3% [230–232] between 1987 and 2006, predominantly due to *P. falciparum* malaria. Due to the low prevalence of malaria and low associated mortality in Europe, no study has been performed to identify risk factors for death or poor long-term prognosis specifically in children in the context of best available supportive care. Therefore, definitions of SM and risk factors have been extrapolated from endemic settings. In the UK, surveillance data showed that children, pregnant women, and older people were particularly at risk of SM, but mortality was highest in those over 65 y of age with very few deaths in children [232,233]. Factors associated with more severe disease include high levels of parasitemia, peripheral *P. falciparum* blood schizonts, pigment deposits in leucocytes, metabolic acidosis, male sex, delay in seeking treatment, diagnosis, or receiving care, nonuse, or inappropriate use of chemoprophylaxis, coma, and renal impairment [232,233].

## Conclusion

Severe childhood malaria is a multi-system disease that encompasses a range of clinical complications including CM, SMA, and respiratory distress. The defining features of SM differ considerably in their frequency, the strength of evidence for their association with adverse outcomes, and their predictive value. Many studies have combined multiple features into prediction models and scoring systems, which has improved the accuracy of prediction and increased ease of use. The most consistent and important predictors of death appear to be coma, markers of metabolic derangement (acidosis and hypoglycemia), and renal failure.

Numerous molecular biomarkers have been proposed to predict outcome in SM, including PfHRP2 concentrations, *var* gene expression profiles, sTREM-1, sFlt-1, and angiopoietins 1 and 2. However, their potential may be compromised by technical challenges to implementation in clinical tests (*var* genes), difficulty in identifying universal thresholds across different transmission settings (PfHRP2), and incomplete understanding of complex interactions with other host response proteins (cytokines). To overcome the latter, feature selection techniques could be implemented to identify the best panels of non-redundant biomarkers and clinical features that together give more accurate outcome predictions. Few studies have included validation of prediction in external cohorts, but this is essential in order to provide evidence that they may be generalizable.

It is clear that prognostic factors and biomarkers specific for distinct SM syndromes also hold promise, and may result in better prediction of adverse outcomes than general prognostic markers in SM. This parallels ongoing debate about whether syndrome-specific adjunctive treatments for SM are needed, essentially applying the principles of personalized medicine to malaria. This approach to prognosis appears most promising in CM, and additional work is needed to identify and validate syndrome-specific biomarkers in SMA and respiratory distress. Perhaps more importantly, there is a large gap between the identification of prognostic features and biomarkers and providing evidence that their measurement contributes to improved short or long-term outcomes for patients. Identification of predictors of neurological sequelae for CM now needs to be combined with intervention studies to prevent or mitigate the effects of these sequelae.

In the ongoing search for predictors of outcome in malaria, we believe that it is essential to consider how biomarkers and other predictors could be used in malaria endemic settings. A successful predictive test is likely to be cheap, rapid, and easy to measure with minimal requirement for infrastructure or specialized training. It should be applicable across different settings, not confounded by the age or immune status of the patient, and it should be highly accurate. Ideally, the results of the prognostic test would guide realistic treatment choices for each individual patient, benefitting those at most risk of death or long-term morbidity.

In this literature review, we have critically evaluated the usefulness and potential of the clinical and molecular features associated with SM mortality and long-term sequelae. However, there is still a need for a systematic meta-analysis of the impact of all these features on prognosis prediction accounting for the heterogeneity between the associated studies. This heterogeneity includes the differences in the sample sizes, study settings, and age range of the patients studied, making a systematic meta-analysis extremely challenging.

## Abbreviations

AOR: Adjusted Odds Ratio
AUC: Area Under the Curve
AUROC: Area Under the Receiver Operating Characteristic
CI: Confidence Interval
CM: Cerebral Malaria
CSF: Cerebrospinal Fluid
EEG: Electroencephalography
EPCR: Endothelial Protein C Receptor
Hb: Haemoglobin
HIV: Human Immunodeficiency Virus
HMGB1: High-Mobility Group Protein-1
HPLA: Hydroxyphenyllactic Acid
HR: Hazards Ratio
IL: Interleukin
IP10: Interferon gamma-induced protein-10
LODS: Lambaréné Organ Dysfunction Score
MRI: Magnetic Resonance Imaging
OR: Odds Ratio
PEDIA: Paediatric Early Death Index for Africa
PfEMP1: *P. falciparum* Erythrocyte Membrane Protein 1
PfHRP2: *P. falciparum* Histidine Rich Protein-2
RR: Relative Risk
sFlt-1: Soluble Vascular Endothelial Growth Factor Receptor 1
SMA: Severe Malarial Anaemia
SMAC: Severe Malaria in African Children
sTREM-1: Soluble form of Triggering Receptor Expressed on Myeloid cells-1
TGF-β: Transforming Growth Factor beta
TNF: Tumour Necrosis Factor
UM: Uncomplicated Malaria
VEGF: Vascular Endothelial Growth Factor
VWF: Von Willebrand factor
WHO: World Health Organisation
